# Misconceptions and Rumors about Ebola Virus Disease in Sub-Saharan Africa: A Systematic Review

**DOI:** 10.3390/ijerph19084714

**Published:** 2022-04-13

**Authors:** Basilua Andre Muzembo, Ngangu Patrick Ntontolo, Nlandu Roger Ngatu, Januka Khatiwada, Tomoko Suzuki, Koji Wada, Kei Kitahara, Shunya Ikeda, Shin-Ichi Miyoshi

**Affiliations:** 1 Graduate School of Medicine, Dentistry and Pharmaceutical Sciences, Okayama University, Okayama 700-8530, Japan; keikitahara@okayama-u.ac.jp (K.K.); miyos-s@okayama-u.ac.jp (S.-I.M.); 2 Department of Family Medicine and Primary Health, Protestant University of Congo, Kinshasa, Democratic Republic of the Congo; patrickntontolo@yahoo.fr; 3 Institut Médical Evangélique (IME), Kimpese, Democratic Republic of the Congo; 4 Department of Public Health, Kagawa University Faculty of Medicine, Miki-cho 761-0793, Japan; ngatu@med.kagawa-u.ac.jp; 5 Social Work Institute, Nakhu-4, Kathmandu, Nepal; j.janukakhatiwada@gmail.com; 6 Department of Public Health, School of Medicine, International University of Health and Welfare, Narita 286-8686, Japan; tsuzuki7@iuhw.ac.jp (T.S.); kwada@iuhw.ac.jp (K.W.); shunya@iuhw.ac.jp (S.I.); 7 Collaborative Research Center of Okayama University for Infectious Diseases in India, Kolkata 700010, India

**Keywords:** Ebola, knowledge, attitudes, practices, beliefs, misperceptions, rumors, sub-Saharan Africa

## Abstract

We sought to summarize knowledge, misconceptions, beliefs, and practices about Ebola that might impede the control of Ebola outbreaks in Africa. We searched Medline, EMBASE, CINAHL, and Google Scholar (through May 2019) for publications reporting on knowledge, attitudes, and practices (KAP) related to Ebola in Africa. In total, 14 of 433 articles were included. Knowledge was evaluated in all 14 articles, and they all highlighted that there are misconceptions and risk behaviors during an Ebola outbreak. Some communities believed that Ebola spreads through the air, mosquito bites, malice from foreign doctors, witchcraft, and houseflies. Because patients believe that Ebola was caused by witchcraft, they sought help from traditional healers. Some people believed that Ebola could be prevented by bathing with salt or hot water. Burial practices where people touch Ebola-infected corpses were common, especially among Muslims. Discriminatory attitudes towards Ebola survivors or their families were also prevalent. Some Ebola survivors were not accepted back in their communities; the possibility of being ostracized from their neighborhoods was high and Ebola survivors had to lead a difficult social life. Most communities affected by Ebola need more comprehensive knowledge on Ebola. Efforts are needed to address misconceptions and risk behaviors surrounding Ebola for future outbreak preparedness in Africa.

## 1. Introduction

Ebola virus disease (EVD or Ebola) outbreaks are complex public health issues and yet many important aspects including their socio-anthropological perspectives need a thorough understanding. Ebola is more commonly known as Ebola hemorrhagic fever. It remains a constant public health problem in sub-Saharan Africa. Ebola was first recognized in 1976 in the Democratic Republic of Congo (DRC) and South Sudan [1]. It is a highly pathogenic disease caused by the Ebola virus (a filovirus) and was named after the Ebola River in northern DRC [1].

Ebola is transmitted to humans through contact with blood or bodily fluids from an infected animal or patient. When Ebola is transmitted to humans, the average case fatality rate is 50% (range, 25–90%) [1]. The main clinical symptoms include acute fever along with gastrointestinal symptoms, fatigue, hypovolemia, electrolyte abnormalities, and in most severe cases Ebola often results in organ failure. The disease can lead to sequelae including ophthalmic manifestations (such as uveitis), stroke, migraine headaches, and persistence of Ebola virus in semen leading to sexual transmission [2,3,4].

Ebola occurrence had been most frequent across the Central Africa Region including the DRC, Republic of Congo, Gabon, Uganda, and Côte d’Ivoire. Up until 2013, it was often treated as a neglected African disease [5]. Ebola became a threat to international biosecurity after decimating approximately 11,322 people in West Africa (Guinea, Liberia, Mali, Nigeria, and Sierra Leone) between 2013 and 2016 [1]. In DRC, 3481 cases of Ebola and 2299 deaths have been reported during 2018–2020 [1]. In order to highlight the significance of Ebola, the World Health Organization (WHO) declared Ebola outbreaks as a public health emergency of international concern in August 2014 (during the 2014–2016 outbreak in West Africa) and in July 2019 (during the 2018–2020 outbreak in DRC).

It is crucial for a given community to have accurate knowledge about mode of transmission, possess good attitude, and observe correct practices for the prevention and control of Ebola. During Ebola outbreaks, the African communities have often been described as “ignorant” [6,7] because of their attachment to cultural beliefs and practices that may enhance Ebola transmission. Some cultural beliefs and practices in sub-Saharan Africa have been associated with increased incidence of Ebola transmission [7,8]. Historically, secret and unsafe funeral and burial practices are common during an Ebola outbreak in some parts of Africa, and they contribute to spread Ebola in an explosive way [9,10]. In addition, some communities in Africa had more trust in traditional healers, religious leaders, and other social leaders as compared to governments even prior to the emergence of Ebola and this belief had been further reinforced during Ebola outbreaks. The knowledge building of these leaders about Ebola may help to control the spread of the disease if they encourage safe burial practices in their communities [11].

Knowledge, attitudes, and practices (KAP) surveys help to identify misconceptions that are potential obstacles to the activities to be implemented and prevent behavioral changes [12]. Even though Ebola is a complex problem, the African people’s KAP on Ebola has the potential to help develop tailored health education programs that are crucial to guide response efforts during Ebola outbreaks.

Thus, we sought to synthesize the evidence on knowledge, misconceptions, rumors, beliefs, and practices about Ebola that might be obstacles in the pathway of controlling Ebola outbreaks in Africa. Therefore, we gathered existing evidence on KAP about Ebola in sub-Saharan Africa communities.

## 2. Methods

### 2.1. Study Design

We undertook a systematic review of studies that investigated KAP of Ebola in sub-Saharan Africa to identify rumors and misconceptions that may constitute obstacles to Ebola containment efforts. We followed the guidelines from preferred reporting items for systematic reviews and meta-analysis (PRISMA) [13]. This systematic review was not registered.

### 2.2. Data Sources and Searches

We searched Medline, EMBASE, CINAHL, and Google Scholar to identify articles related to Ebola KAP among sub-Saharan African communities. The search was based on studies published in English from 1976 to May 2019. The following combined keywords were used: (‘Ebola’ OR ‘Ebola virus’ OR ‘EBOV’ OR ‘Ebola virus disease’ OR ‘EVD’ OR ‘Ebola virus disease epidemic’ OR ‘EVD epidemic’ OR ‘Ebola hemorrhagic fever’ OR ‘Ebola fever epidemic’ OR ‘Ebola virus outbreak’) AND (‘knowledge’ OR ‘attitude’ OR ‘practice’ OR ‘perceptions’ OR ‘beliefs’) AND (‘sub-Saharan Africa’ OR ‘Africa’ OR ‘West Africa’ OR ‘country name (Democratic Republic Of Congo, Republic of Congo, Uganda, Gabon, Sudan, Sierra Leone, Liberia, Ghana, Guinea, Senegal, Mali, Nigeria, and Cote d’Ivoire)’. To identify additional articles, we conducted a manual search using the reference lists of the selected studies.

### 2.3. Eligibility Criteria

To be included, a study had to meet the following inclusion criteria: be published as a peer-reviewed article (Study type), be carried out in people living in Africa (Population), and assess KAP towards Ebola or awareness about Ebola (Exposure). It was not considered compulsory for a study to include a comparison group (Comparison). We focused on features of culture and beliefs as well as human practices that spread Ebola and any local perspective that may amplify the transmission of Ebola (Outcomes).

We excluded studies solely related to a specific group of individuals (e.g., healthcare workers or medical students) and those clearly outside of our scope (for example, reviews, editorials, opinions, and studies not carried out in Africa).

### 2.4. Study Selection

After exporting the retrieved records to Endnote (version X7) software (Thomson Reuters, Carlsbad, CA, USA), two researchers (BAM and NRN) performed the initial title screening of the retrieved records. First, we removed duplicate articles and all those that were not relevant to our study. Then, titles and abstracts of the retrieved studies were screened for inclusion by BAM and NRN.

### 2.5. Data Extraction

Before data extraction, we conceived a standardized chart that we used for data extraction.

For data extraction, it was performed by two researchers (BAM and NRN) separately. Any disagreements were resolved by consensus and further reading of the article. Extracted information included the KAP elements, the method of questionnaire administration, the year of publication, the year of data collection, the study location and its design, the sample size, and the authors’ main conclusion.

### 2.6. Assessment of Study Quality and Data Synthesis

Two reviewers (BAM and NRN) independently evaluated the reporting methodology of included studies. For qualitative studies, we used a modified Critical Appraisal Skills Programme (CASP) tool [14] to assess the strengths and limitations of the methodology.

For studies using quantitative methods, the quality of reporting was assessed based on the completeness of information disclosed to the reader using a modified checklist from Downes and colleagues [15]. We developed a checklist containing information related to the objective or aim of the study, study design, sample size, description of the study population, validation of KAP questionnaire, sufficient description of methods, ethical clearance, reporting results, and discussion on relevance and limitations. A score of “1” was given for each reported item. Scores were classified as high (8–10), moderate (5–7), or of low quality (≤4).

Results were synthesized narratively.

## 3. Results

### 3.1. Study Selection and Description of Studies

**Figure 1:**
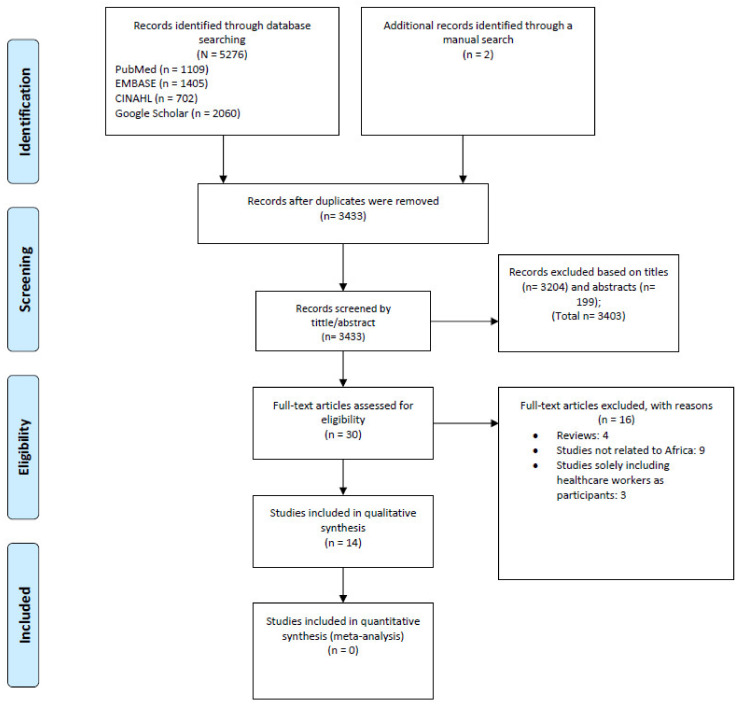
Flow chart summarizing study evidence search and selection.

**Figure 2:**
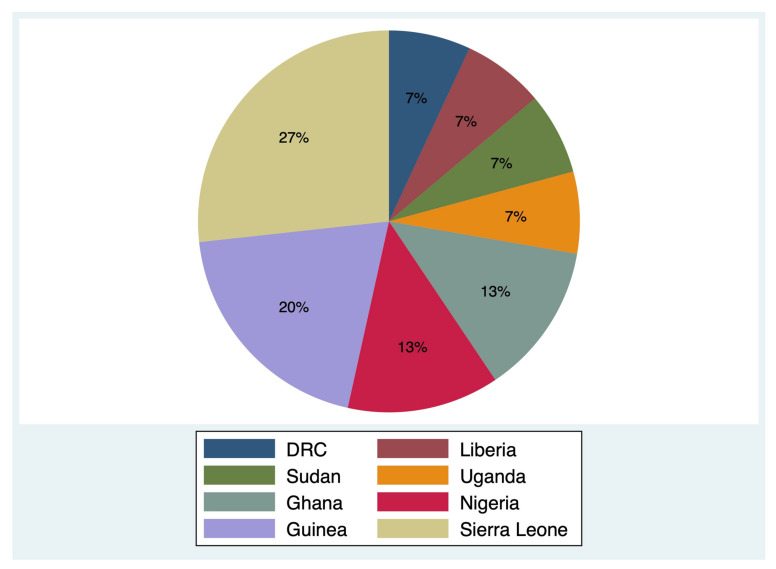
Country of origin of the included studies (*n* = 14). DRC = Democratic Republic of the Congo.

We retrieved 5278 articles through our search in databases. Two additional articles were retrieved through a manual search of the reference lists of the selected articles. Of them, 30 articles were potentially eligible and only 14 met inclusion criteria for this review [16,17,18,19,20,21,22,23,24,25,26,27,28,29]. Detailed study selection is displayed in Figure 1. The reviewed studies (Table 1) were from eight sub-Saharan African countries, i.e., DRC (*n* = 1) [16], Guinea (*n* = 3) [19,20,26], Ghana (*n* = 2) [18,24], Liberia (*n* = 1) [28], Nigeria (*n* = 2) [25,27], Sudan (*n* = 1) [22], Sierra Leone (*n* = 4) [17,19,21,23], and Uganda (*n* = 1) [29] (Figure 2).

Out of the 14 reviewed studies, only one was a qualitative study [24], and the 13 others were quantitative studies with cross-sectional designs. However, the study conducted in DRC used a mixed-methods approach including quantitative and qualitative data [16]. Sample sizes ranged from 253 to 10,604 participants (30,829 participants in total), with two studies reported as being nationwide surveys [20,21]. In most of the studies, the authors developed their own questionnaire, and two studies did not specify the source of the questionnaire [20,28].

**Figure 3:**
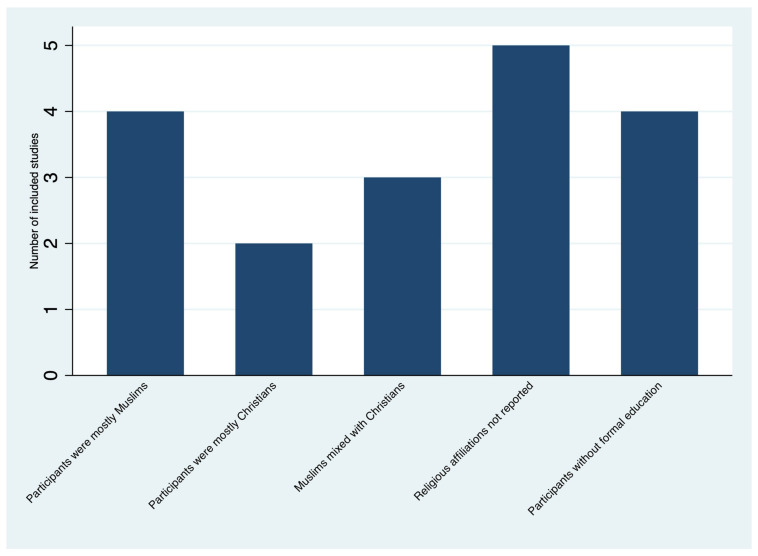
Selected socio-demographic data of the included studies (*n* = 14).

Four studies reported including participants with no formal education ranging from 18% in DRC [16], 26% (in Sierra Leone) [21], and 50% (in Guinea and Sierra Leone) [19,20]. Nine of the included studies reported religious affiliations of the participants (Figure 3), but it was not reported in five studies [16,22,25,27,28]. In some studies, most of the participants were Muslims [17,19,21,25], Christians [20,27], or a codominance of Christians and Muslims [18,23,24].

**Table 1:** Characteristics of included studies.

First Author (Year)	Study Design	Setting	Study Population (Focus Groups)	Age (Year)	Sample Size	KAP Element	Data Collection Instrument	Data Collection Period	Study Quality (Score) *
Claude (2018) [16]	CS and qualitative research	DRC	Community, IDPs, pygmy, and HCWs	≥25 and 15–24	582	K; A; P	Prepared by the authors with reference to previously published KAP	2018	High (9)
Winters (2018) [17]	CS	Sierra Leone	Community	21–35	10,604	K; A; P	Prepared by the authors	2014–2015	High (9)
Tenkorang (2018) [18]	CS	Ghana	Community	18–69	800	K; P	Prepared by the author	2016	High (9)
Jalloh (2017) [21]	CS	Sierra Leone	Community	≥25 and 15–24	1413	K; A; P	Prepared by the authors with reference to previously published KAP	2014	High (10)
Jalloh (2017) [20]	CS	Guinea	Community	≥25 and 15–24	6273	K; A; P	NR	2015	High (9)
Jalloh (2017) [19]	CS	Sierra Leone and Guinea	Community	35–40	1137	K; A; P	Prepared by the authors with reference to previously published KAP	2015	High (8)
Mohamed (2017) [22]	CS	Sudan	Community	>18	1255	K; A; P	Prepared by the authors	2015	High (8)
Nyakarahuka (2017) [29]	CS	Uganda	Community	33	740	K; A	Prepared by the authors	2015	High (10)
Jiang (2016) [23]	CS	Sierra Leone	Community	NR	466	K; A; P	Prepared by the authors	2015	Moderate (7)
Adongo ** (2016) [24]	Qualitative research	Ghana	Community and nurses	NR	235	K; A	Prepared by the authors	2015	-
Iliyasu (2015) [25]	CS	Nigeria	Community and HCWs	32	1035	K; A; P	Prepared by the authors with reference to previously published KAP	2014	High (9)
Buli (2015) [26]	CS	Guinea	Community	≥25 and 18–24	358	K; A; P	Prepared by the authors	2014–2015	Moderate (7)
Gidado (2015) [27]	CS	Nigeria	Community	34	5322	K; A; P	Prepared by the authors	2014	Moderate (7)
Kobayashi (2015) [28]	CS	Liberia	Community	32	609	K; A; P	NR	2014	High (8)

CS = cross-sectional; KAP = knowledge, attitudes, practices; HCWs = healthcare workers; NR = not reported; DRC = Democratic Republic of Congo; IDPs = internally displaced persons. * A score “1” was given for each reported item. Scores were classified as high (8–10), moderate (5–7), or of low quality (≤4): see Supplementary Table S1 for details. ** Critical Appraisal Skills Programme tool was used for quality assessment (see Supplementary Table S2 for details).

### 3.2. Methodological Assessment of Included Studies

In cross-sectional studies, the reporting scores ranged from 7 to 10 out of 10. It was rated as high in 10 studies and as moderate in 3 studies (Supplementary Table S1).

In addition, studies including qualitative data were deemed to have minor limitations concerning the reporting quality (Supplementary Table S2). No study was excluded because of poor quality.

### 3.3. Knowledge, Awareness, and Rumors about EBOLA

The reviewed studies revealed that most participants had heard of Ebola (Table 2) and sometimes awareness reached 100% [21]. However, concerns were evident in specific knowledge gaps on the etiology of Ebola, symptoms, transmission risk, prevention, and treatment (Table 3). More specifically, reviewed studies found that some participants were unaware of the causative agent of Ebola or its transmission mode [21,22,24,25,26,27,28,29]. For instance, results from Sierra Leone showed that 60–75% of participants did not know that Ebola could be transmitted through human-to-human contact [19,21]. In Sudan, 50% of participants were unaware of the causative agent of Ebola and 30% were unaware of its transmission mode [22]. Only 29% of participants in Sudan knew that direct contact with infected bodily fluids can transmit Ebola [22]. In the Kano state of Nigeria, 37% of participants did not know that avoiding contact with the dead remains of an infected individual could prevent Ebola [25]. In DRC, 11% of the participants were unaware of human-to-human transmission of Ebola via an infected corpse and only 1% of the internally displaced persons had comprehensive knowledge of Ebola [16].

In Uganda and Liberia, some participants believed that Ebola is maliciously spread by foreign aid doctors/workers [29] or brought into Africa by white people [28]. Denial of an Ebola outbreak is common in the affected communities. Some participants believed that Ebola was not real. For example, some people still believed that they were not at risk of contracting Ebola even though the epidemic was present [23]. In Guinea, some participants (23.8%) were unaware that Ebola existed in their community although the disease was recorded in their prefecture and had already claimed lives [26]. In Nigeria, there were participants (61%) who believed that they could not contract the disease because they were under spiritual and divine protection [27].

Although there is consistent evidence that hemorrhagic signs may be absent in Ebola patients, participants still expected bleeding from body orifices to be symptoms of Ebola [29]. Information on the necessary precautions that should be taken to avoid Ebola infection and Ebola symptoms (i.e., distinguishing it from a disease with similar symptoms such as malaria) was desired by the respondents [21,24]. Educational level was found to influence knowledge about Ebola [22,25,26,27,29]. Respondents with sound knowledge were more likely to have good Ebola preventive behaviors [18,25].

### 3.4. Attitudes, Beliefs, and Misconceptions

Each study reported some misconceptions regarding Ebola, although the degree of misconceptions varied across the studies (Table 3). The main misconceptions were about Ebola transmission and management. For instance, there were beliefs amongst participants that Ebola could be spread through the air [16,20,21,22,24,25,28,29], and through mosquito bites [16,19,21,25,29], sorcery or witchcraft [20,26,29], and houseflies [24]. For instance, 49% [20] to 61% [19] of participants in Guinea believed that mosquito bites can transmit Ebola. In Sudan, 39% of participants reported that Ebola can be transmitted through the air or a vector route [22].

In addition, 36% of participants in Guinea [26] and 8–31% of the participants in Sierra Leone/Guinea [19] believed that Ebola was God’s punishment for their sins. A similar belief was also found in Ghana [24]. Ebola was perceived to be prevented by bathing with salt and hot water [16,20,21,25,26]. For instance, 41% of participants in Sierra Leone believed that bathing with salt and hot water can prevent Ebola [21]. Some people believed that Ebola could be treated by spiritual healers or prayers [19,21,24,26,27] or traditional healers [19,22,26,28]. For instance, in Sierra Leone, 19% of participants believed that Ebola can be treated by spiritual healers, whereas 5% believed that it can be cured by traditional healers [21]. Furthermore, in Sudan, 75% of participants believed that traditional healers could cure the disease [22].

Participants in Nigeria also believed that drinking salty water and eating bitter kola could treat Ebola [25,27]. Furthermore, most of the studies reported an overall discriminatory attitude towards Ebola survivors [19,20,21,24,28,29]. Reports from Guinea and Sierra Leone indicated that Ebola survivors were not accepted into their communities [19,20,21]. Their integration into the community was also difficult. For instance, people were afraid to buy groceries from Ebola-cured patients [19,20,21]. Ebola patients suffered from being ostracized by their neighborhoods. A study from Uganda reported that 40% of participants had a negative attitude towards Ebola such as refusing treatment from health workers [29].

### 3.5. Practices on Ebola Prevention and Treatment

Some participants considered traditional burial rituals and funeral practices to be high-risk practices that exacerbate Ebola transmission, i.e., washing and handling the corpse of an Ebola victim or touching Ebola-affected corpses during funerals [16,20,21,22,24,27]. For instance, in DRC, 89% of the participants knew of the human-to-human transmission of Ebola through dead bodies [16]. Despite this high awareness about human-to-human transmission of Ebola, 8% stated that they would wash and handle the corpse of a family victim [16]. A further 10% of participants indicated that they would refuse burial performed by trained teams [16]. In Nigeria, 94% of the participants did not know that participation in the burial rites of an Ebola-deceased patient could help spread Ebola [27]. In Soudan, 37% of villagers did not perceive the risk of an Ebola-affected corpse [22]. In Guinea, 3% reported that they would touch and wash an Ebola-affected corpse [20]. The effect of religion on Ebola-preventive behaviors was also highlighted; Muslins were less likely to engage in Ebola-preventive behaviors [18]. Muslims were less likely to accept alternative burial methods that do not involve touching or washing dead bodies [18,19].

Intention to hide Ebola cases in homes and fear of Ebola treatment units are common across sub-Saharan Africa [16,19,28]; the reason for this is that they would be unable to see their family members or they would die if their family members visited an Ebola treatment unit, or simply fear of being stigmatized. For instance, 17% of participants in DRC reported an intention to hide Ebola patients in their homes [16]. Furthermore, only 14% of participants in Guinea and 5% in Sierra Leone indicated that they would use a health facility if a family member was suspected of having Ebola [19].

Several reviewed studies found that the use of radios was the preferred method for communities to obtain the information they need to protect themselves against Ebola [17,19,21,22,23,27,29].

In Sierra Leone [21], information about Ebola from healthcare professionals was more trusted than information from the media [21]. Frequent hand hygiene was recommended in communities faced with Ebola, but it may not be done correctly. The Nigeria KAP survey found that only 2.2% of the respondents washed all parts of their hand when they were asked to demonstrate their hand-washing practices [27].

## 4. Discussion

In this review, we sought to summarize knowledge limitation, misconceptions, rumors, beliefs, and practices that might impede the control of Ebola outbreaks in sub-Saharan Africa. An in-depth understanding of these issues will make Ebola response teams better prepared in the event of future Ebola outbreaks. Although studies used different KAP questionnaires, findings were consistent and they could be summarized in the following five principal themes: (1) limited biomedical knowledge about Ebola, (2) perceived causes of Ebola that are beyond the bio-medical science paradigm and intention to choose unorthodox treatment, (3) reduced trust in health authorities, and hospitals were seen as a place to die, (4) high-risk behaviors due to unsafe funeral/burial practices, and (5) stigma.

### 4.1. Limited Biomedical Knowledge about Ebola

We found evidence that many participants had heard of Ebola, and most of them were quite aware of its danger, despite having limited knowledge of Ebola transmission and prevention. Some participants highlighted that they were less aware of how to protect themselves against probable Ebola death cases that might happen at home (home care provided prior to death). This knowledge gap was also reported in a study conducted in Sierra Leone [30], which highlighted the community’s lack of knowledge about the necessary precautions to self-safeguard. For example, healthy individuals providing care to an Ebola-suspected sick family member without any appropriate protective clothing. There are several possible explanations for the limited knowledge on Ebola among the African people. One explanation could be the limited access to Ebola-related updates and information. All the previous outbreaks have generally occurred in remote areas. However, major cities were also affected during the 2014–2016 Ebola outbreak in West Africa. Since some remote areas in Africa have limited access to electricity, the internet, and media channels, it is plausible that this situation might also hinder some governments from providing information on Ebola.

In addition, there is evidence that educational background can also be a reason for the limited knowledge. Hence, the extrapolation of the limited knowledge to all African communities affected by Ebola might not be correct. Some of the reviewed studies included participants without any formal education which could also be the reason behind the participants’ limited knowledge about Ebola. Educated individuals may be better informed about Ebola and possess high risk perceptions of contracting Ebola, but level of education alone may not be enough to induce behavioral changes needed to prevent the spread of Ebola. Poverty, civil conflicts, and inadequate preparation of fragile health systems could also indirectly contribute to the knowledge limitation about Ebola among African populations [31].

Many of the participants showed a desire to understand the difference between the symptoms of Ebola and other tropical diseases such as malaria, typhoid fever, and cholera, which are relatively more prevalent in sub-Saharan Africa. Lack of awareness could delay the process of healthcare services to the sick individual and that might prove fatal. For common ailments, people in sub-Saharan communities opt either for self-medication or over-the-counter medicine from private drug shops [32]. As a consequence, they might not seek timely care until the aggravation of Ebola symptoms.

### 4.2. Perceived Causes of Ebola That Are beyond the Bio-Medical Science Paradigm and Intention to Choose Unorthodox Treatment

Most of the information about Ebola transmission has been reported by Osterholm et al. [33] and does not support the views or beliefs of Ebola transmission that are beyond the bio-medical science paradigm. Many of the participants in the reviewed studies held incorrect beliefs about Ebola. For instance, some of them perceived Ebola as a disease from God to punish people. Further, the perceptions of witchcraft or sorcery as a cause of Ebola were popular among participants and were consistent with previous studies [11,34]. These views and beliefs often emerge during an Ebola outbreak and are widespread among people living in sub-Saharan Africa [35]. Misconceptions and beliefs in non-biomedical explanations of Ebola are shared among community members or in social networks. The consequences are that they can promote resistance against control measures. For instance, once people are sick, they might not seek formal medical care or be reluctant to be vaccinated.

Reviewed studies also showed that Ebola was believed to be cured by prayers, spiritual healers, or traditional healers. It is important to note that in many sub-Saharan communities, local concepts regarding the cause of a disease determine what steps those people take next, concerning where to go for treatment. A considerable section of the African population is pious and believes that supernatural spirits are responsible for diseases along with biological agents. This encourages them to seek divine intervention in the case of unfamiliar diseases such as Ebola, where conventional medicines are not very efficient. In such a case, traditional healers are the last resort because they offer treatments which will not hamper the cultural and traditional beliefs of the community in question. Other possible explanations for consulting traditional healers during Ebola outbreaks include poverty (hospital care seems to be too expensive) and distrust in health authorities or health systems (advance payment for providing treatment) [11].

### 4.3. Reduced Trust in Health Authorities, and Hospitals Seen as a Place to Die

Reduced trust in healthcare authorities or systems might be due to poor infrastructure and insufficient resources of most health systems in sub-Saharan Africa [31]. In fact, during Ebola outbreaks, the response team often argues that it is there to protect the health of the people. However, the response team might not provide enough services for the less fatal diseases such as malaria. This makes people distrust healthcare authorities [36]. In addition, a long history of endemic corruption, unmet social needs, and weak governments had also been noted to erode trust [11]. For instance, some people in the DRC believed that Ebola was spread by the Armed Forces as they are involved in outbreak responses [37]. We surmised that armed conflict and insecurity [16,38] that lead to violence against health workers may also explain the reduced trust in health authorities [38].

Other culprits are foreign aid workers, who are also believed to be accountable for the reduced trust. These foreign aid workers provide treatments to Ebola patients in treatment centers, where most of the time these patients die in the presence of unfamiliar people [39]. This may also explain in part the reduced utilization of hospitals in some counties during Ebola. Furthermore, when community members and friends are not allowed to properly honor the deceased Ebola victim, it may reinforce feelings of mistrust towards health workers.

### 4.4. High-Risk Behaviors Due to Unsafe Funeral/Burial Practices

In this study, some people stated that they would make physical contact with a deceased Ebola victim as a gesture of affection. Other reports have also shown that for the sake of power transfer, people in sub-Saharan Africa may sleep with infected Ebola corpses or bathe with water used to wash Ebola corpses [40], making it easy for the Ebola virus to spread. Touching, washing, and redressing a deceased person is a common cultural practice in sub-Saharan Africa and have a great significance for the African people. It is critical to note that Ebola deaths that occur in the community constitute a source of infection. This is because people involved do not know that the deceased died of Ebola. Therefore, people would touch the deceased and become infected. This cultural practice varies with religion, geography, and the death of prominent individuals in the community. For example, a well-known male Guinean midwife assistant who regularly performed circumcision in the community died probably due to Ebola, but the cause of his death was unknown during the funeral. His funeral attendance was linked to 85 confirmed secondary cases of Ebola [41]. The ritual of providing physical contact with the dead has been promoted by African religious leaders for centuries, including washing the deceased. Reviewed studies reported that Muslims were less likely to accept alternative burial methods [18,19]. In West Africa, burial ritual is considered as pious. Therefore, residents are likely to refuse alternative burial methods, such as cremation [42]. The acceptance rate of burial alternatives to cultural practices can differ within the same country. For example, the people in southern and eastern regions of Sierra Leone, where Ebola cases were found low were found to avoid unsafe burials. This is in contrast to western and northern regions where Ebola cases were high [43]. It is also important to note that family members are afraid of the stigma if a loved one is not given a safe and dignified burial involving community members [44].

### 4.5. Stigma

Lack of treatment and a high mortality rate might explain the fear and stigma associated with Ebola. This finding has also been highlighted in other epidemics such as HIV/AIDS [45,46]. Therefore, the lessons learned from the HIV-related stigma can be applied to eradicate the stigma associated with Ebola such as accurate health information campaigns and community mobilization. For Ebola survivors, people in the community may believe that they are still infectious. Therefore, Ebola survivors and their families have a fear of being rejected by their communities, and being removed from housing and work [47]. Participation of survivors in the prevention and control activities can help in the fight against the Ebola stigma [48,49].

### 4.6. Recommendations

Improving someone’s knowledge is crucial to enhance positive attitudes as it can result in behavioral changes [50]. This needs support from health authorities who should bring clear and consistent messages. Several approaches had been used to improve the knowledge of Ebola in sub-Saharan communities which include tailored health education, constant engagement of communities involving religious leaders [11,48], and medical students [51].

We suggest that the response teams who guide the community during Ebola outbreaks identify possible misconceptions and take those misperceptions into account and incorporate them into response efforts. Denying the above perceptions of the disease can hamper Ebola containment efforts [52]. Therefore, any response against Ebola should be modeled by considering African culture.

In addition, traditional and spiritual healers are usually held in high regard in some African communities. Therefore, traditional and spiritual healers are important mediators and should be encouraged to refer Ebola suspects to hospitals. To improve referral of patients from traditional/spiritual healers to hospitals, several approaches are advocated including increased collaboration between healthcare professionals, and training the traditional and spiritual healers on Ebola symptoms [42]. Building trust with the local community involving local partners (such as religious leaders and traditional healers) has been highlighted to be of critical importance in the fight against Ebola [53].

During Ebola outbreaks, it is crucial to promote safe burials, i.e., corpses must be handled by a trained burial team. This should be done with dignity and respect to the deceased and their families [54]. The absence of proper respect to the deceased person may promote secret unsafe burials, under-reporting Ebola suspects and all this will contribute to Ebola transmission. Examples of dignified manners include the involvement of community members in the burials, respecting customs, and marking names on the graves [54]. The offering of final prayer by a religious leader and allowing family members to observe the burial from a safe distance were cited as an alternative to traditional burials [19].

Limitations of this review include the possible information bias related to gaps between what participants have reported in primary studies and what the participants do in real life. For example, people may touch an Ebola-infected body and yet deny that they do so [30]. This is a common limitation with KAP surveys [12]. Despite this fact, all available information on misconceptions and rumors was summarized. It is clear that this study provides important information to the scientific community, i.e., a summary of beliefs and practices that may impede control efforts during future Ebola outbreaks in Africa. Our study found only 14 articles in the peer-reviewed literature; this may have been due to the fact that articles published in French and gray literature were not searched, which we consider as a limitation of our study. These limitations would not change the importance of considering the influence of socio-cultural aspects during any response to Ebola outbreaks.

## 5. Conclusions

This review provides evidence that most communities affected by Ebola in sub-Saharan Africa need more comprehensive knowledge on Ebola. Local Ebola responses may face several difficulties including those related to socio-cultural beliefs, high-risk practices, rumors, and misconceptions. This study emphasizes that KAP data during an Ebola outbreak provide important information to build up health education messages tailored to levels of Ebola knowledge in the community. Efforts are needed to address concerns, misconceptions, and myths surrounding Ebola in order to be prepared for future outbreaks in Africa.

## Figures and Tables

**Table 2:** Summary of authors’ conclusion.

First Author, Year	KAP Element	Questionnaire	Authors’ Conclusion
Claude (2018) [16]	K; A; P	Open- and close-ended, and focus group discussion	High knowledge on transmission. However, respondents would practice traditional burials involving physical contact with a family member corpse; hide family members from health authorities. Knowledge among IDPs was low. Armed conflict impeded control efforts in eastern DRC.
Winters (2018) [17]	K; A; P	Open- and close-ended	Exposure to information sources was associated with higher knowledge and protective behaviors. Misconceptions and risk behavior were also prevalent.
Tenkorang (2018) [18]	K; P	Close-ended	High level of Ebola knowledge and awareness. However, misconceptions remained present.
Jalloh (2017) [21]	K; A; P	Open- and close-ended	Awareness of Ebola was high. However, misconceptions and stigma towards Ebola survivors were common.
Jalloh (2017) [20]	K; A; P	Open- and close-ended	Awareness of the cause of Ebola, its transmission, and prevention was high. However, nearly half of participants believed that Ebola could be transmitted by air or through mosquito bites. Stigma towards Ebola survivors was also prevalent.
Jalloh (2017) [19]	K; A; P	Open- and close-ended	High knowledge on prevention. However, some respondents endorsed stigma towards Ebola survivors.
Mohamed (2017) [22]	K; A; P	Open- and close-ended	Poor knowledge, a fair attitude, and suboptimal practices on Ebola.
Nyakarahuka (2017) [29]	K; A	Close-ended	Moderate knowledge about EVD and 60% of respondents had a positive towards practices to prevent and control Ebola.
Jiang (2016) [23]	K; A; P	Close-ended	After training, knowledge was high, and attitudes related to prevention was satisfactory. However, symptoms and transmission modes needed public education.
Adongo (2016) [24]	K; A	Focus group discussion and semi structured in-depth interviews	High level of Ebola knowledge and awareness. However, misconceptions on transmission were present. Potential stigma towards people who might be infected with Ebola or work with Ebola patients.
Iliyasu (2015) [25]	K; A; P	Self-administered	Ebola-related KAP was at suboptimal levels. However, myths and misconceptions remained present
Buli (2015) [26]	K; A; P	Close-ended	High level of Ebola awareness. However, comprehensive knowledge about Ebola was low. Misconceptions remained present.
Gidado (2015) [27]	K; A; P	Close-ended	Existence of gap in Ebola knowledge and perception. Misconceptions and stigma towards Ebola survivors were also prevalent.
Kobayashi (2015) [28]	K; A; P	Close-ended	Awareness of Ebola was high. However, knowledge of symptoms of Ebola was poor and stigma towards Ebola survivors and Ebola treatment units were common

CS = cross-sectional; EVD = Ebola virus disease; KAP = knowledge, attitudes, practices; IDPs = internally displaced persons; DRC = Democratic Republic of Congo.

**Table 3:** Summary of gaps in knowledge, misconceptions and rumors.

Knowledge Gaps, Misconceptions and Rumors	Number of Studies, *n* (%)	Countries (% of Participants) *
Unaware of the transmission mode	10 (71)	DRC (11%) [16], Ghana (-) [24], Sierra Leone (60–75%) [19,21], Guinea (10%) [26], Liberia (7–35%) [28], Nigeria (27%) [25], Sudan (30%) [22], and Uganda (49%) [29]
A person could contract Ebola from the air	8 (57)	DRC (-) [16], Ghana (-) [24], Guinea (27%) [20], Guinea (26%) [26], Nigeria (12–42%) [25], Sierra Leone (19%) [19], Sudan (39%) [22], and Uganda (17%) [29]
Mosquito bites or houseflies can transmit Ebola	6 (43)	DRC (-) [16], Ghana (-) [24], Guinea (61%) [19], Guinea (49%) [20], Guinea (27%) [26], and Uganda (11%) [29]
Intending to touch a suspected corpse or attending a traditional burial	6 (43)	DRC (10%) [16], Ghana (17%) [18], Guinea (38%) [19], Nigeria (37%) [25], Sierra Leone (19%) [19], and Sudan (21%) [22]
Stigma towards Ebola survivors	6 (43)	Ghana (-) [24], Guinea (11%) [19], Liberia (59%) [28], Nigeria (64%) [27], Sierra Leone (9%) [19], and Uganda (53%) [29]
Bathing with salt or hot water can prevent Ebola	6 (43)	DRC (-) [16], Guinea (13%) [19], Sierra Leone (17%) [19], Guinea (22%) [20], Guinea (46%) [26], Nigeria (6%) [27], and Sierra Leone (41%) [21]
Prayers or spiritual healers can cure Ebola	6 (43)	Ghana (-) [24], Guinea (3%) [19], Sierra Leone (1%) [19], Guinea (5%) [20], Guinea (9%) [26], Nigeria (57%) [25], and Sierra Leone (19%) [21]
Traditional healers can cure Ebola	5 (36)	Guinea (15%) [26], Liberia (95%) [28], Guinea (3%) [19], Sierra Leone (1%) [19], Sierra Leone (5%) [21] and Sudan (75%) [22]
Ebola seen as a punishment from God	4 (29)	Ghana (-) [24], Guinea (36%) [26], Guinea (31%) [19], Sierra Leone (8%) [19], and Sierra Leone (8%) [21]
Hide Ebola cases in homes	3 (21)	DRC (17%) [16], Liberia (9%) [28], Guinea (86%) [19], and Sierra Leone (95%) [19]
Ebola might be spread by witchcraft	3 (21)	Guinea (9%) [26] and Uganda (1%) [29]
Foreign aid workers spread Ebola	2 (14)	Liberia (12%) [28] and Uganda (-) [29]
Ebola could be cured by drinking salty water or eating bitter kola nut	2 (14)	Nigeria (93%) [25] and Nigeria (-) [27]
Believing that Ebola cannot infect them because of divine protection	1 (7)	Nigeria (61%) [27]
Not aware of Ebola in the community	1 (7)	Guinea (24%) [26]

DRC = Democratic Republic of Congo. * In some studies, the percentage of participants was not reported or could not be extracted from figures.

## Data Availability

All relevant data are within the manuscript and its supporting information files.

## Supplementary Materials

The following supporting information can be downloaded at: https://www.mdpi.com/article/10.3390/ijerph19084714/s1, Table S1: Quality assessment of included studies (*n* = 13); Table S2: Methodological assessment of included studies with qualitative design using a modified CASP tool (*n* = 3).

## References

[1] World Health Organization (WHO) (2021). Ebola Virus Disease. https://www.who.int/news-room/fact-sheets/detail/ebola-virus-disease.

[2] Vetter P., Kaiser L., Schibler M., Ciglenecki I., Bausch D.G. (2016). Sequelae of Ebola virus disease: The emergency within the emergency. Lancet Infect. Dis..

[3] Clark D.V., Kibuuka H., Millard M., Wakabi S., Lukwago L., Taylor A., Eller M.A., Eller L.A., Michael N.L., Honko A.N. (2015). Long-term sequelae after Ebola virus disease in Bundibugyo, Uganda: A retrospective cohort study. Lancet Infect. Dis..

[4] Mate S.E., Kugelman J.R., Nyenswah T.G., Ladner J.T., Wiley M.R., Cordier-Lassalle T., Christie A., Schroth G.P., Gross S.M., Davies-Wayne G.J. (2015). Molecular evidence of sexual transmission of Ebola Virus. N. Engl. J. Med..

[5] Awah P.K., Boock A.U., Kum K.A. (2015). Ebola Virus Diseases in Africa: A commentary on its history, local and global context. Pan Afr. Med. J..

[6] Gray N., Stringer B., Bark G., Heller Perache A., Jephcott F., Broeder R., Kremer R., Jimissa A.S., Samba T.T. (2018). When Ebola enters a home, a family, a community: A qualitative study of population perspectives on Ebola control measures in rural and urban areas of Sierra Leone. PLoS Negl. Trop. Dis..

[7] Leach M. (2010). Time to put Ebola in context. Interview with Dr Melissa Leach. Bull. World Health Organ..

[8] Roca A., Afolabi M.O., Saidu Y., Kampmann B. (2015). Ebola: A holistic approach is required to achieve effective management and control. J. Allergy Clin. Immunol..

[9] Coltart C.E., Lindsey B., Ghinai I., Johnson A.M., Heymann D.L. (2017). The Ebola outbreak, 2013–2016: Old lessons for new epidemics. Philos. Trans. R. Soc. Lond. B Biol. Sci..

[10] Hersey S., Martel L.D., Jambai A., Keita S., Yoti Z., Meyer E., Seeman S., Bennett S., Ratto J., Morgan O. (2015). Ebola Virus Disease—Sierra Leone and Guinea, August 2015. Morb. Mortal. Wkly. Rep..

[11] Muzembo B.A., Ntontolo N.P., Ngatu N.R., Khatiwada J., Ngombe K.L., Numbi O.L., Nzaji K.M., Maotela K.J., Ngoyi M.J., Suzuki T. (2020). Local perspectives on Ebola during its tenth outbreak in DR Congo: A nationwide qualitative study. PLoS ONE.

[12] United States Agency for International Development (USAID) The KAP Survey Model (Knowledge, Attitudes, and Practices). https://www.spring-nutrition.org/publications/tool-summaries/kap-survey-model-knowledge-attitudes-and-practices.

[13] Moher D., Shamseer L., Clarke M., Ghersi D., Liberati A., Petticrew M., Shekelle P., Stewart L.A., Group P.-P. (2015). Preferred reporting items for systematic review and meta-analysis protocols (PRISMA-P) 2015 statement. Syst. Rev..

[14] Critical Appraisal Skills Programme (2018). CASP Qualitative Checklist. https://casp-uk.b-cdn.net/wp-content/uploads/2018/03/CASP-Qualitative-Checklist-2018_fillable_form.pdf.

[15] Downes M.J., Brennan M.L., Williams H.C., Dean R.S. (2016). Development of a critical appraisal tool to assess the quality of cross-sectional studies (AXIS). BMJ Open.

[16] Claude K.M., Underschultz J., Hawkes M.T. (2018). Ebola virus epidemic in war-torn eastern DR Congo. Lancet.

[17] Winters M., Jalloh M.F., Sengeh P., Jalloh M.B., Conteh L., Bunnell R., Li W., Zeebari Z., Nordenstedt H. (2018). Risk communication and Ebola-specific knowledge and behavior during 2014–2015 outbreak, Sierra Leone. Emerg. Infect. Dis..

[18] Tenkorang E.Y. (2018). Effect of knowledge and perceptions of risks on Ebola-preventive behaviours in Ghana. Int. Health.

[19] Jalloh M.F., Sengeh P., Monasch R., Jalloh M.B., DeLuca N., Dyson M., Golfa S., Sakurai Y., Conteh L., Sesay S. (2017). National survey of Ebola-related knowledge, attitudes and practices before the outbreak peak in Sierra Leone: August 2014. BMJ Glob. Health.

[20] Jalloh M.F., Robinson S.J., Corker J., Li W., Irwin K., Barry A.M., Ntuba P.N., Diallo A.A., Jalloh M.B., Nyuma J. (2017). Knowledge, attitudes, and practices related to Ebola virus disease at the end of a national epidemic—Guinea, August 2015. MMWR Morb. Mortal. Wkly. Rep..

[21] Jalloh M.F., Bunnell R., Robinson S., Jalloh M.B., Barry A.M., Corker J., Sengeh P., VanSteelandt A., Li W., Dafae F. (2017). Assessments of Ebola knowledge, attitudes and practices in Forecariah, Guinea and Kambia, Sierra Leone, July–August 2015. Philos. Trans. R. Soc. Lond. B Biol. Sci..

[22] Mohamed M.M.G., Shwaib H.M., Fahim M.M., Ahmed E.A., Omer M.K., Monier I.A., Balla S.A. (2017). Ebola hemorrhagic fever under scope, view of knowledge, attitude and practice from rural Sudan in 2015. J. Infect. Public Health.

[23] Jiang H., Shi G.Q., Tu W.X., Zheng C.J., Lai X.H., Li X.X., Wei Q., Li M., Deng L.Q., Huo X. (2016). Rapid assessment of knowledge, attitudes, practices, and risk perception related to the prevention and control of Ebola virus disease in three communities of Sierra Leone. Infect. Dis. Poverty.

[24] Adongo P.B., Tabong P.T., Asampong E., Ansong J., Robalo M., Adanu R.M. (2016). Beyond knowledge and awareness: Addressing misconceptions in Ghana’s preparation towards an outbreak of Ebola virus disease. PLoS ONE.

[25] Iliyasu G., Ogoina D., Otu A.A., Dayyab F.M., Ebenso B., Otokpa D., Rotifa S., Olomo W.T., Habib A.G. (2015). A Multi-site knowledge attitude and practice survey of Ebola virus disease in Nigeria. PLoS ONE.

[26] Buli B.G., Mayigane L.N., Oketta J.F., Soumouk A., Sandouno T.E., Camara B., Toure M.S., Conde A. (2015). Misconceptions about Ebola seriously affect the prevention efforts: KAP related to Ebola prevention and treatment in Kouroussa Prefecture, Guinea. Pan Afr. Med. J..

[27] Gidado S., Oladimeji A.M., Roberts A.A., Nguku P., Nwangwu I.G., Waziri N.E., Shuaib F., Oguntimehin O., Musa E., Nzuki C. (2015). Public knowledge, perception and source of information on Ebola virus disease—Lagos, Nigeria; September, 2014. PLoS Curr..

[28] Kobayashi M., Beer K.D., Bjork A., Chatham-Stephens K., Cherry C.C., Arzoaquoi S., Frank W., Kumeh O., Sieka J., Yeiah A. (2015). Community knowledge, attitudes, and practices regarding Ebola virus disease—Five counties, Liberia, September–October, 2014. MMWR Morb. Mortal. Wkly. Rep..

[29] Nyakarahuka L., Skjerve E., Nabadda D., Sitali D.C., Mumba C., Mwiine F.N., Lutwama J.J., Balinandi S., Shoemaker T., Kankya C. (2017). Knowledge and attitude towards Ebola and Marburg virus diseases in Uganda using quantitative and participatory epidemiology techniques. PLoS Negl. Trop. Dis..

[30] Stehling-Ariza T., Rosewell A., Moiba S.A., Yorpie B.B., Ndomaina K.D., Jimissa K.S., Leidman E., Rijken D.J., Basler C., Wood J. (2016). The impact of active surveillance and health education on an Ebola virus disease cluster—Kono District, Sierra Leone, 2014–2015. BMC Infect. Dis..

[31] Buseh A.G., Stevens P.E., Bromberg M., Kelber S.T. (2015). The Ebola epidemic in West Africa: Challenges, opportunities, and policy priority areas. Nurs. Outlook.

[32] Hodel E.M., Kabanywanyi A.M., Malila A., Zanolari B., Mercier T., Beck H.P., Buclin T., Olliaro P., Decosterd L.A., Genton B. (2009). Residual antimalarials in malaria patients from Tanzania—Implications on drug efficacy assessment and spread of parasite resistance. PLoS ONE.

[33] Osterholm M.T., Moore K.A., Kelley N.S., Brosseau L.M., Wong G., Murphy F.A., Peters C.J., LeDuc J.W., Russell P.K., Van Herp M. (2015). Transmission of Ebola viruses: What we know and what we do not know. MBio.

[34] Kasereka M.C., Hawkes M.T. (2019). The cat that kills people: Community beliefs about Ebola origins and implications for disease control in Eastern Democratic Republic of the Congo. Pathog. Glob. Health.

[35] Oduyemi R.O., Ayegboyin M., Salami K.K. (2016). Perceptions of Ebola virus disease in Nigeria: Understanding the influence of imagination on health orientation. Int. J. Nurs. Pract..

[36] Dhillon R.S., Kelly J.D. (2015). Community Trust and the Ebola Endgame. N. Engl. J. Med..

[37] Oppenheim B., Lidow N., Ayscue P., Saylors K., Mbala P., Kumakamba C., Kleinman M. (2019). Knowledge and beliefs about Ebola virus in a conflict-affected area: Early evidence from the North Kivu outbreak. J. Glob. Health.

[38] Vinck P., Pham P.N., Bindu K.K., Bedford J., Nilles E.J. (2019). Institutional trust and misinformation in the response to the 2018–19 Ebola outbreak in North Kivu, DR Congo: A population-based survey. Lancet Infect. Dis..

[39] Wells C.R., Pandey A., Ndeffo Mbah M.L., Gauzere B.A., Malvy D., Singer B.H., Galvani A.P. (2019). The exacerbation of Ebola outbreaks by conflict in the Democratic Republic of the Congo. Proc. Natl. Acad. Sci. USA.

[40] Parker M., Hanson T.M., Vandi A., Babawo L.S., Allen T. (2019). Ebola, community engagement, and saving loved ones. Lancet.

[41] World Health Organization (WHO) (2015). Factors That Contributed to Undetected Spread of the Ebola Virus and Impeded Rapid Containment. https://www.who.int/csr/disease/ebola/one-year-report/factors/en.

[42] Victory K.R., Coronado F., Ifono S.O., Soropogui T., Dahl B.A., Centers for Disease Control and Prevention (CDC) (2015). Ebola transmission linked to a single traditional funeral ceremony—Kissidougou, Guinea, December, 2014–January 2015. MMWR Morb. Mortal. Wkly. Rep..

[43] Manguvo A., Mafuvadze B. (2015). The impact of traditional and religious practices on the spread of Ebola in West Africa: Time for a strategic shift. Pan Afr. Med. J..

[44] Lyons P., Winters M., Zeebari Z., Schmidt-Hellerau K., Sengeh P., Jalloh M.B., Jalloh M.F., Nordenstedt H. (2021). Engaging religious leaders to promote safe burial practices during the 2014–2016 Ebola virus disease outbreak, Sierra Leone. Bull. World Health Organ..

[45] Lee-Kwan S.H., DeLuca N., Bunnell R., Clayton H.B., Turay A.S., Mansaray Y. (2017). Facilitators and barriers to community acceptance of safe, dignified medical burials in the context of an Ebola epidemic, Sierra Leone, 2014. J. Health Commun..

[46] Mahajan A.P., Sayles J.N., Patel V.A., Remien R.H., Sawires S.R., Ortiz D.J., Szekeres G., Coates T.J. (2008). Stigma in the HIV/AIDS epidemic: A review of the literature and recommendations for the way forward. AIDS.

[47] Drain P.K. (2015). Ebola: Lessons learned from HIV and tuberculosis epidemics. Lancet Infect. Dis..

[48] Lo T.Q., Marston B.J., Dahl B.A., De Cock K.M. (2017). Ebola: Anatomy of an epidemic. Annu. Rev. Med..

[49] Nuriddin A., Jalloh M.F., Meyer E., Bunnell R., Bio F.A., Jalloh M.B., Sengeh P., Hageman K.M., Carroll D.D., Conteh L. (2018). Trust, fear, stigma and disruptions: Community perceptions and experiences during periods of low but ongoing transmission of Ebola virus disease in Sierra Leone, 2015. BMJ Glob. Health.

[50] James P.B., Wardle J., Steel A., Adams J. (2019). Post-Ebola psychosocial experiences and coping mechanisms among Ebola survivors: A systematic review. Trop. Med. Int. Health.

[51] Glanz K., Bishop D.B. (2010). The role of behavioral science theory in development and implementation of public health interventions. Annu. Rev. Public Health.

[52] Masumbuko Claude K., Hawkes M.T. (2020). Ebola crisis in Eastern Democratic Republic of Congo: Student-led community engagement. Pathog. Glob. Health.

[53] Sabuni L.P. (2007). Dilemma with the local perception of causes of illnesses in central Africa: Muted concept but prevalent in everyday life. Qual. Health Res..

[54] Nielsen C.F., Kidd S., Sillah A.R., Davis E., Mermin J., Kilmarx P.H., Centers for Disease Control and Prevention (CDC) (2015). Improving burial practices and cemetery management during an Ebola virus disease epidemic—Sierra Leone, 2014. MMWR Morb. Mortal. Wkly. Rep..

