# Supplementary material for: Misconceptions and Rumors about Ebola Virus Disease in Sub-Saharan Africa: A Systematic Review
Source: Int J Environ Res Public Health. 2022 Apr 13;19(8):4714. doi: 10.3390/ijerph19084714 (PMC9027331; doi:10.3390/ijerph19084714)
Supplement: Supplementary file 1 [file ijerph-19-04714-s001.zip › ijerph-1669009-supplementary.pdf]

Supplementary Materials

Supplementary Table S1. Quality assessment of included studies (n = 13).

	Introduction				Methods			Results	Discussion		
First author (year)	Objectives clearly defined	Study design explained	Sample size justified	Target population clearly defined	Outcome variables measured correctly using instruments that had been piloted or previously published	Methods (including statistical methods) sufficiently reported	Ethical clearance reported	Results reported in accordance with the described methods	Results relevance discussed	Limitations discussed	Score
Claude (2018) [16]	1	1	0	1	1	1	1	1	1	1	9
Winters (2018)[17]	1	1	0	1	1	1	1	1	1	1	9
Tenkorang (2018)[18]	1	1	0	0	1	0	1	0	0	0	9
Jalloh (2017)[21]	1	1	1	1	1	1	1	1	1	1	10
Jalloh (2017)[20]	1	1	1	1	1	1	0	1	1	1	9
Jalloh (2017)[19]	1	1	0	1	0	1	1	1	1	1	8
Mohamed (2017)[22]	1	1	0	1	1	1	1	1	1	0	8
Nyakarahuka (2017)[29]	1	1	1		1	1	1	1	1	1	10
Jiang (2016)[23]	1	1	0	1	0	0	1	1	1	1	7
Iliyasu (2015)[25]	1	1	0	1	1	1	1	1	1	1	9
Buli (2015)[26]	1	1	1	1	0	1	1	1	0	0	7
Gidado (2015)[27]	1	1	1	1	0	1	0	1	1	0	7
Kobayashi (2015)[28]	1	1	0	1	1	1	1	1	1	0	8

A score "1" was given for each reported item. Scores were classified as high (8-10), moderate (5-7), or of low quality (≤4).

Supplementary Table S2. Methodological assessment of included studies with qualitative design using a modified CASP tool (n = 3).

First author (year)	**Was There a Clear Statement of the Aims of the Research?**	**Is a Qualitative Methodology Appropriate?**	**Was the Research Design Appropriate to Address the Aims of the Research?**	**Was the Recruitment Strategy Appropriate to the Aims of the Research?**	**Was the Data Collected in a Way that Addressed the Research Issue?**	**Have Ethical Issues Been Taken into Consideration?**	**Was the Data Analysis Sufficiently Rigorous?**	**Is There a Clear Statement of Findings?**	**Is the Research Valuable?**
Adongo (2016)[24]	Yes	Yes	Yes	Yes	Yes	Yes	Yes	Yes	Yes
Claude (2018)[16]	Yes	Yes	Yes	Yes	Yes	Yes	Yes	Yes	Yes

CASP= Critical Appraisal Skills Programme

References

16. Claude, K.M.; Underschultz, J.; Hawkes, M.T. Ebola virus epidemic in war-torn eastern DR Congo. *Lancet* **2018**, *392*, 1399–1401, doi:10.1016/s0140-6736(18)32419-x.
17. Winters, M.; Jalloh, M.F.; Sengeh, P.; Jalloh, M.B.; Conteh, L.; Bunnell, R.; Li, W.; Zeebari, Z.; Nordenstedt, H. Risk communication and Ebola-specific knowledge and behavior during 2014–2015 outbreak, Sierra Leone. *Emerg. Infect. Dis.* **2018**, *24*, 336–344. https://doi.org/10.3201/eid2402.171028.
18. Tenkorang, E.Y. Effect of knowledge and perceptions of risks on Ebola-preventive behaviours in Ghana. *Int. Health* **2018**, *10*, 202–210. https://doi.org/10.1093/inthealth/ihy009.
19. Jalloh, M.F.; Sengeh, P.; Monasch, R.; Jalloh, M.B.; DeLuca, N.; Dyson, M.; Golfa, S.; Sakurai, Y.; Conteh, L.; Sesay, S.; et al. National survey of Ebola-related knowledge, attitudes and practices before the outbreak peak in Sierra Leone: August 2014. *BMJ Glob. Health* **2017**, *2*, e000285. https://doi.org/10.1136/bmjgh-2017-000285.
20. Jalloh, M.F.; Robinson, S.J.; Corker, J.; Li, W.; Irwin, K.; Barry, A.M.; Ntuba, P.N.; Diallo, A.A.; Jalloh, M.B.; Nyuma, J.; et al. Knowledge, attitudes, and practices related to Ebola virus disease at the end of a national epidemic—Guinea, August 2015. *MMWR Morb. Mortal. Wkly. Rep.* **2017**, *66*, 1109–1115. https://doi.org/10.15585/mmwr.mm6641a4.
21. Jalloh, M.F.; Bunnell, R.; Robinson, S.; Jalloh, M.B.; Barry, A.M.; Corker, J.; Sengeh, P.; VanSteelandt, A.; Li, W.; Dafae, F.; et al. Assessments of Ebola knowledge, attitudes and practices in Forecariah, Guinea and Kambia, Sierra Leone, July–August 2015. *Philos. Trans. R. Soc. Lond. B Biol. Sci.* **2017**, *372*, 20160304. https://doi.org/10.1098/rstb.2016.0304.
22. Mohamed, M.M.G.; Shwaib, H.M.; Fahim, M.M.; Ahmed, E.A.; Omer, M.K.; Monier, I.A.; Balla, S.A. Ebola hemorrhagic fever under scope, view of knowledge, attitude and practice from rural Sudan in 2015. *J. Infect. Public Health* **2017**, *10*, 287–294. https://doi.org/10.1016/j.jiph.2016.05.016.
23. Jiang, H.; Shi, G.Q.; Tu, W.X.; Zheng, C.J.; Lai, X.H.; Li, X.X.; Wei, Q.; Li, M.; Deng, L.Q.; Huo, X.; et al. Rapid assessment of knowledge, attitudes, practices, and risk perception related to the prevention and control of Ebola virus disease in three communities of Sierra Leone. *Infect. Dis. Poverty* **2016**, *5*, 53. https://doi.org/10.1186/s40249-016-0142-9.
24. Adongo, P.B.; Tabong, P.T.; Asampong, E.; Ansong, J.; Robalo, M.; Adanu, R.M. Beyond knowledge and awareness: Addressing misconceptions in Ghana's preparation towards an outbreak of Ebola virus disease. *PLoS ONE* **2016**, *11*, e0149627. https://doi.org/10.1371/journal.pone.0149627.

25. Iliyasu, G.; Ogoina, D.; Otu, A.A.; Dayyab, F.M.; Ebenso, B.; Otokpa, D.; Rotifa, S.; Olomo, W.T.; Habib, A.G. A Multi-site knowledge attitude and practice survey of Ebola virus disease in Nigeria. *PLoS ONE* **2015**, *10*, e0135955. https://doi.org/10.1371/journal.pone.0135955.
26. Buli, B.G.; Mayigane, L.N.; Oketta, J.F.; Soumouk, A.; Sandouno, T.E.; Camara, B.; Toure, M.S.; Conde, A. Misconceptions about Ebola seriously affect the prevention efforts: KAP related to Ebola prevention and treatment in Kouroussa Prefecture, Guinea. *Pan Afr. Med. J.* **2015**, *22* (Suppl. 1), 11. https://doi.org/10.11694/pamj.supp.2015.22.1.6269.
27. Gidado, S.; Oladimeji, A.M.; Roberts, A.A.; Nguku, P.; Nwangwu, I.G.; Waziri, N.E.; Shuaib, F.; Oguntimehin, O.; Musa, E.; Nzuki, C.; et al. Public knowledge, perception and source of information on Ebola virus disease—Lagos, Nigeria; September, 2014. *PLoS Curr.* **2015**, *7*, ecurrents.outbreaks.0b805cac244d700a47d6a3713ef2d6db. https://doi.org/10.1371/currents.outbreaks.0b805cac244d700a47d6a3713ef2d6db.
28. Kobayashi, M.; Beer, K.D.; Bjork, A.; Chatham-Stephens, K.; Cherry, C.C.; Arzoaquoi, S.; Frank, W.; Kumeh, O.; Sieka, J.; Yeiah, A.; et al. Community knowledge, attitudes, and practices regarding Ebola virus disease—Five counties, Liberia, September–October, 2014. *MMWR Morb. Mortal. Wkly. Rep.* **2015**, *64*, 714–718.
29. Nyakarahuka, L.; Skjerve, E.; Nabadda, D.; Sitali, D.C.; Mumba, C.; Mwiine, F.N.; Lutwama, J.J.; Balinandi, S.; Shoemaker, T.; Kankya, C. Knowledge and attitude towards Ebola and Marburg virus diseases in Uganda using quantitative and participatory epidemiology techniques. *PLoS Negl. Trop. Dis.* **2017**, *11*, e0005907. https://doi.org/10.1371/journal.pntd.0005907.
